# Determinants of Telemedicine Satisfaction in Inflammatory Bowel Disease Patients: A Multi-Centre Cross-Sectional Study

**DOI:** 10.3390/medicina62010147

**Published:** 2026-01-12

**Authors:** Piergiorgio Martella, Alessio Lo Cascio, Arianna Povoli, Luca Molino, Giovanni Cangelosi, Nicoletta Orgiana, Stefano Mancin, Federica Tomassini, Giuseppina Martino, Stefano Martino, Fabrizio Bossa, Valentin Calvez, Gabriele Rumi, Franco Scaldaferri, Daniele Napolitano

**Affiliations:** 1 Neuroscience Department, Neurosurgery Care Management, Gemelli IRCCS University Hospital Foundation, Largo Agostino Gemelli 8, 00168 Rome, Italy; piergiorgio.martella@policlinicogemelli.it; 2 Direction of Health Professions, La Maddalena Cancer Center, Via San Lorenzo 312, 90146 Palermo, Italy; locascio.alessio@lamaddalenanet.it; 3 Department of Gastroenterology, Azienda Sanitaria Universitaria Friuli Centrale, 33100 Udine, Italy; ariannapovoli@gmail.com; 4 School of Medicine, Faculty of Medicine, Università Cattolica del Sacro Cuore, Largo Francesco Vito 1, 00168 Rome, Italy; luca.molino01@icatt.it; 5 School of Pharmacy, Polo Medicina Sperimentale e Sanità Pubblica “Stefania Scuri”, University of Camerino, 62032 Camerino, Italy; 6 CUT, Fondazione Policlinico Gemelli IRCCS, 00168 Rome, Italy; nicoletta.orgiana@policlinicogemelli.it; 7 IRCCS Humanitas Research Hospital, Via Manzoni 56, 20089 Rozzano, Italy; 8 Fondazione Policlinico Universitario Agostino Gemelli IRCCS, 00168 Rome, Italy; federica.tomassini@policlinicogemelli.it; 9 Division of Gastroenterology and Endoscopy, Fondazione IRCCS “Casa Sollievo della Sofferenza”, Viale Cappuccini 1, 71013 San Giovanni Rotondo, Italy; giusy.martino1969@gmail.com (G.M.); f.bossa@operapadrepio.it (F.B.); 10 ASL Foggia, 71121 Foggia, Italy; step170790@hotmail.it; 11 IBD Unit, Digestive Disease Center (CeMAD), Department of Translational Medicine and Surgery, Fondazione Policlinico Universitario Agostino Gemelli IRCCS, 00168 Rome, Italy; valentino.calvez@gmail.com (V.C.); gabriele.rumi@policlinicogemelli.it (G.R.); franco.scaldaferri@policlinicogemelli.it (F.S.); daniele.napolitano@policlinicogemelli.it (D.N.); 12 Internal Medicine, Division of Internal Medicine and Gastroenterology, Fondazione Policlinico Universitario Agostino Gemelli IRCCS, 00168 Rome, Italy; 13 SITRA—Scientific Direction, Fondazione Policlinico Universitario Agostino Gemelli IRCCS, 00168 Rome, Italy

**Keywords:** telemedicine, inflammatory bowel disease, patient satisfaction, chronic disease management, Italian multicentre study

## Abstract

*Background and Objectives:* Telemedicine has become an essential component of chronic Inflammatory Bowel Disease (IBD) care, yet the factors that shape patient satisfaction with remote consultations remain only partially understood. This study aimed to assess satisfaction with institutional telemedicine services among Italian patients with ulcerative colitis (UC) and Crohn’s disease (CD), and to identify sociodemographic, clinical and organisational predictors to inform more person-centred telehealth models. *Materials and Methods:* We conducted a prospective, multi-centre, cross-sectional study in three IBD units in northern, central and southern Italy between June and October 2024. Consecutive adult patients who had completed a scheduled, non-emergency telemedicine visit were invited within 24–48 h to complete an online questionnaire including the Italian Telemedicine Satisfaction Questionnaire (I-TSQ), sociodemographic items, IBD-related variables, and telemedicine process indicators (accessibility, technology usability, technical support, time saved). Data were analysed descriptively and with multivariable linear regression to determine independent predictors of satisfaction, adjusting for recruiting centre. *Results:* A total of 705 patients participated (54.9% UC; 55.3% disease duration > 10 years). Overall, telemedicine satisfaction was high (mean I-TSQ total 57.5 ± 4.9; range 35–70), and all respondents reported reduced indirect costs compared with in-person visits. Greater ease of technology use, more frequent contact with the care team, male sex, older age, and employment were independently associated with higher satisfaction scores. Conversely, first-ever teleconsultations, CD, subcutaneous therapies, more difficult platform access, and the need for technical support were linked to lower satisfaction. Model fit was modest (R^2^ up to 0.20), suggesting the presence of additional unmeasured relational and contextual factors. *Conclusions:* Telemedicine for IBD is widely accepted in Italy, but satisfaction is strongly conditioned by digital usability, previous experience, and clinical complexity. Tailored telehealth pathways that incorporate user-friendly platforms, proactive technical support, and attention to vulnerable subgroups are needed to translate high satisfaction into sustained, equitable remote care.

## 1. Introduction

Inflammatory Bowel Disease (IBD), comprising ulcerative colitis (UC) and Crohn’s disease (CD), has an unpredictable, relapsing, and often debilitating course [1]. The continuous need for follow-up, clinical monitoring, and multidisciplinary support, typical of chronic conditions, creates major challenges for healthcare systems, particularly where specialist centers are unevenly distributed [2,3,4]. In this setting, telemedicine, defined as the remote delivery of healthcare via information and communication technologies, has emerged as a promising strategy to improve access, continuity, and efficiency [5,6,7].

Since the pandemic, the role of telemedicine in chronic care has been highlighted across multiple settings [8]. Reviews on IBD and health technologies report that telemedicine interventions may improve quality of life, help maintain remission and reduce relapse, support treatment adherence, and decrease hospital visits [9,10]. Evidence also suggests that remote IBD care is non-inferior to conventional management in terms of disease-related hospitalizations, while users value reduced travel and wait times [11]. Web-based interventions, virtual clinics, smartphone applications, and teleconsulting can facilitate self-management, enable early treatment optimization, and promote better clinical outcomes, including in low-volume districts [7,12]. However, the overall clinical effectiveness of telemedicine in IBD remains uncertain [13].

Beyond IBD, experience from other chronic conditions offers useful insights: recent reviews show that most patients evaluate telehealth positively, with gains in self-care skills, self-monitoring behaviors, and clinical outcomes [14,15,16,17]. In a mixed chronic-conditions cohort, 62.9% of users reported satisfaction with the received telehealth services [18]. In type 2 diabetes, an internet-based U-healthcare integrated management system achieved better glycemic control, reduced HbA1c, lowered triglyceride levels, and improved adherence to medical instructions [19]. Although consensus is not universal, telemedicine appears pivotal for heart failure management and promising for hypertension control [20,21]. Qualitative research further indicates that remote follow-up promotes perceived safety, greater understanding of illness, and a more active patient role [22].

Within IBD specifically, patients report high satisfaction with telemedicine and dedicated platforms, with a strong inclination toward continued telematic or hybrid models due to time and cost savings, reduced travel, and perceived continuity of care [23]. These experience outcomes are accompanied by short-term clinical non-inferiority (remission, quality of life) relative to standard care, suggesting that telemedicine is a sustainable alternative when medical examination is not necessarily required [11,24]. At the same time, satisfaction is likely shaped by age, digital familiarity, disease severity, and psychosocial factors, which can modulate perceptions of the service [25].

Although some studies note associations between clinical factors and satisfaction, the literature lacks a consolidated mapping of satisfaction determinants and a predictive model integrating clinical and technological features [26,27]. Moreover, the direct link between patient satisfaction and subsequent improvements in clinical pathways, treatment adherence, or care burden remains insufficiently explored in IBD [10,27].

In studies evaluating telemedicine services, patient satisfaction is commonly assessed using standardized questionnaires developed according to established psychometric principles [28]. These instruments are typically constructed through iterative processes, including theory-driven item generation, expert content validation, and empirical testing, to ensure adequate reliability, construct validity, and dimensional stability [29]. The use of validated satisfaction measures, particularly those adapted to the linguistic and cultural context of the target population, is essential to ensure methodological rigor and comparability across telemedicine studies [30,31].

This paper aims to address these gaps by conducting a cross-sectional study to evaluate satisfaction among IBD patients who have used telemedicine services and to identify individual predictors of telemedicine that can inform the design of more personalized, effective, and acceptable telehealth models, particularly for complex chronic condition like IBD.

## 2. Materials and Methods

### 2.1. Design

This was a prospective, multicenter, cross-sectional study conducted in Italy between June and October 2024. Three IBD units, located in northern, central, and southern Italy, participated using an identical protocol, the same electronic data collection system, and shared operational definitions. The study was conceived to describe patient satisfaction with institutional telemedicine visits for IBD and to explore sociodemographic, clinical, and organisational factors associated with higher or lower levels of satisfaction in real-world practice. Consecutive patients who had just completed a scheduled telemedicine encounter were invited to participate to capture the experience closest in time to the remote consultation and minimize recall bias. Informed consent was obtained before patient participation in the study. For the questionnaire, patients were sent a link to an online form (Microsoft Office Forms) to complete. The study adhered to the Strengthening the Reporting of Observational Studies in Epidemiology (STROBE) reporting guidelines for the reporting tool (Supplementary File S1) [32,33].

### 2.2. Inclusion and Exclusion Criteria

Patients were eligible if they were 18 years of age or older, had a confirmed diagnosis of UC or CD made by the treating gastroenterologist, had completed a planned (non-emergency) telemedicine visit with one of the participating centres during the study period, were able to read and understand Italian, and provided electronic informed consent before accessing the questionnaire. Patients were excluded if the remote contact was carried out solely for administrative reasons, if the teleconsultation was urgent or unplanned and did not allow for the research invitation, if cognitive, linguistic, or technological limitations prevented autonomous completion of the online form, or if the patient declined to participate. Recruitment was consecutive in all three centres to reflect the real mix of IBD patients followed through telemedicine, irrespective of disease duration, treatment line, or symptom burden.

### 2.3. Instruments

Data were collected through a secure web-based platform, managed at each site by a trained local investigator (an IBD nurse, research nurse, or gastroenterology staff member). Within 24–48 h after the telemedicine visit, eligible patients received a personalized link via email or short message service, along with a brief study description, assurances of confidentiality, and instructions to complete the questionnaire. The 24–48-h timeframe was chosen to ensure that patients were still referring to the specific telemedicine encounter but had enough time to reflect on its usefulness, accessibility, and relational aspects. The questionnaire consisted of four sections for each domain, as reported below:

- Telemedicine satisfaction was assessed using the Italian version of the Telemedicine Satisfaction Questionnaire (I-TSQ), which was adapted and validated from the original Telemedicine Satisfaction Questionnaire (TSQ) developed in Hong Kong [31,32], the original TSQ is a 14-item self-report instrument designed to capture patients’ satisfaction with telemedicine encounters across multiple experiential dimensions, including perceived quality of care, comparability with face-to-face consultations, and quality of interaction with healthcare professionals. Items are rated on a Likert-type scale, with higher scores reflecting greater satisfaction, and the original version demonstrated excellent internal consistency (Cronbach’s α = 0.93) and sound construct validity. The Italian adaptation of the TSQ underwent rigorous psychometric validation, including linguistic and cultural adaptation, confirmatory factor analysis, and reliability testing in a sample of Italian patients with chronic conditions, including inflammatory bowel disease. The validation study confirmed the original theoretical framework, supporting a three-factor structure comprising Quality of Care, Similarity to Face-to-Face Encounter, and Perception of Interaction. Model fit indices indicated good construct validity (Comparative Fit Index = 0.926; Root Mean Square Error of Approximation = 0.047, 90% CI 0.000–0.079), consistent with accepted psychometric standards [31,32]. Reliability estimates for the Italian version were satisfactory across all subscales, with McDonald’s omega coefficients of 0.74 for Quality of Care, 0.80 for Similarity to Face-to-Face Encounter, and 0.75 for Perception of Interaction, as well as high reliability for the overall scale (ω = 0.88). These values indicate adequate internal consistency and support the use of both domain-specific scores and a global satisfaction score. In the present study, domain scores were calculated by summing item responses within each factor. In contrast, the overall I-TSQ score was obtained by summing all 14 items, yielding a total score ranging from 35 to 70. Higher scores indicate greater satisfaction with telemedicine services. Overall, the I-TSQ is a psychometrically robust and contextually appropriate instrument for assessing patient satisfaction with telemedicine among Italian patients with inflammatory bowel disease [32].
- Sociodemographic variables. The electronic form collected Gender, Marital status, educational level, and occupation, to allow description of the sample and to test the influence of these factors on satisfaction.
- Clinical variables. The following IBD-related data were obtained from the clinical record or patient self-report: diagnosis (UC or CD), disease duration (≤1 year, 1–5 years, 6–10 years, >10 years), current treatment (biologic agents, immunosuppressants, 5-ASA or other), presence of current symptoms (abdominal pain, diarrhoea, rectal bleeding, fatigue, or none), and previous surgery.
- Telemedicine process variables. Because organisational and technological aspects are known to affect the patient’s experience, we also collected the reason for the televisit (follow-up, review of tests, therapy renewal, mild flare), perceived ease of access to the telemedicine platform, ease of using the required technology, and the need for technical support during the teleconsultation. Ease of platform access referred to patients’ perceived ability to connect to the telemedicine visit, including login procedures, connection stability, and absence of technical barriers, and was assessed using a 5-point Likert scale ranging from “very difficult” to “very easy.” The need for technical support was defined as requiring assistance from healthcare staff or information technology personnel to complete the televisit due to connection issues, device configuration problems, or platform-related difficulties [34]. In addition, we assessed time saved compared with an in-person visit, perceived contribution of telemedicine to disease management, and whether telemedicine enabled more frequent contact with the clinical team. These variables were included to capture organisational and technological features of the telemedicine pathway, to allow centre-level and subgroup comparisons, and to explore their association with patient-reported satisfaction outcomes.

### 2.4. Data Analysis

All completed questionnaires were exported in anonymised form and analysed using R 4.5.0 software [35]. Categorical variables were summarised as *n* and %, while continuous variables were expressed as mean and standard deviation (SD) or, when non-normally distributed, as median and interquartile range (IQR). To examine the predictors of telemedicine satisfaction, we estimated four multiple linear regression models, corresponding to the three dimensions of the Telemedicine Satisfaction Questionnaire (TSQ)—Quality of Care, Similarity to face-to-face visits, and Perception of Interaction—and one additional model assessing Overall Satisfaction.

Each model included a set of theoretically relevant predictors, selected based on prior literature and conceptual rationale. Independent variables comprised technical support received, occupation, age, first teleconsultation, disease type (CD vs. UC), frequency of teleconsultations, gender, perceived access (Likert 1–5), technology ease of use (Likert 1–5), educational level, treatment type (immunosuppressants, anti-inflammatory drugs, biologics), and therapy route (subcutaneous vs. intravenous). All categorical predictors were dummy-coded with the first group as the reference category (e.g., female, no technical support, UC, intravenous therapy).

The analytic sample included 705 patients with IBD. Given the number of predictors (k = 13) and a significance threshold of α = 0.05, an a priori power analysis for the overall regression F-test was performed. Using conventional parameters, the analysis indicated that with *n* = 705 the design had >0.99 power to detect medium-sized effects (e.g., R^2^ ≥ 0.09). The minimum detectable effect size (MDES) for 80%, 90%, and 95% power corresponded to R^2^ values of 0.026, 0.032, and 0.038, respectively, confirming that the study was well-powered to identify even small effects across models.

All models were estimated using unstandardized coefficients (β) and standard errors (SE). Statistical significance was set at *p* < 0.05. Model fit indices (F and R^2^).

Before analysis, data were screened for missingness, outliers, and assumptions of multiple regression. Variance inflation factors (VIFs) were below 2.0 for all predictors, indicating no issues with multicollinearity. Residuals were approximately normally distributed and displayed no major deviations from homoscedasticity.

Although participating centres were located in northern, central, and southern Italy, analyses were not stratified by geographical area. This choice was guided by the primary objective of identifying individual- and process-level determinants of telemedicine satisfaction, as well as by the limited number of centres per macro-region, which could have introduced centre-specific organizational effects and reduced statistical robustness. To account for contextual variability, recruiting centre was included as an adjustment factor in multivariable models.

### 2.5. Ethical Approval

The study was carried out in accordance with the Declaration of Helsinki (World Medical Association, 2024) and with current Italian regulations on multicenter observational research. The full protocol was reviewed and approved by the Ethics Committee CEL Area 1 of Foggia, protocol number [7 March 2022 N°27/CE.]. Each participating centre adhered to the approved protocol and, where necessary, obtained local authorization before commencing enrollment. Participation was voluntary; patients could access the questionnaire only after providing electronic informed consent on the initial page; data were collected anonymously and stored on password-protected devices accessible only to the research team.

## 3. Results

Descriptives are reported in Table 1. All (100%) the respondents had stated that telemedicine has reduced medical visit costs (transport, work absence, etc.). The study included 705 IBD patients with a mean age of 43.12 (12.32), predominantly female (52.3%) and married (47.7%). Over half had UC (54.9%) and a long disease history (>10 years in 55.3%). Televisits were primarily used to review tests or renew therapy (41.1%); 59.9% were first-time teleconsultations. Regarding organisational and technological aspects, approximately nine out of ten patients reported that accessing the telemedicine platform was easy or very easy, indicating minimal perceived barriers to participating in the remote visit. Similarly, ease of technology use was rated as easy or very easy by the large majority of respondents, suggesting high overall usability of the telemedicine system.

Almost all participants (99.0%) reported receiving adequate technical support. Importantly, this variable reflects the availability of assistance when needed rather than the absence of technical difficulties and may therefore capture differences in patients’ digital confidence rather than service quality alone. Most patients (79.3%) reported that telemedicine enabled more frequent contact with the clinical team, and all participants (100%) confirmed a reduction in indirect costs compared with in-person visits.

### 3.1. Satisfaction Levels

Satisfaction with telemedicine was high across domains (Table 2). Mean scores were 16.85 for Quality of Care, 20.21 for Similarity to face-to-face visits, and 16.59 for Perception of Interaction; overall satisfaction reached 57.50 (SD 4.93) on a 35–70 scale, confirming good acceptance of the remote model.

### 3.2. Predictors of Telemedicine Satisfaction

Table 3 reports the multivariable predictors of TSQ domains and overall satisfaction. Across all models, receipt of adequate technical support was a strong negative predictor of patient-reported outcomes, with significant associations for Quality of Care (β = −2.75, *p* < 0.001), Similarity (β = −4.60, *p* < 0.001), Perception of Interaction (β = −2.54, *p* < 0.001), and Overall Satisfaction (β = −10.04, *p* < 0.001). Conversely, ease of technology use was consistently and positively associated with all outcomes (βs = 0.41–1.75, all *p* ≤ 0.05). Patients attending their first teleconsultation reported significantly lower scores across domains (e.g., Quality of Care: β = −0.66, *p* < 0.001; Overall Satisfaction: β = −1.98, *p* < 0.01). Older age was associated with higher ratings for Perception of Interaction (β = 0.016, *p* < 0.001) and Overall Satisfaction (β = 0.026, *p* < 0.05). Additionally, male gender was linked to more favorable evaluations across outcomes (βs = 0.32–1.20, all *p* ≤ 0.05). Clinical characteristics showed mixed effects: patients with Crohn’s disease consistently reported lower scores compared to those with UC (e.g., Perception of Interaction: β = −0.64, *p* < 0.001), while those on subcutaneous therapies reported significantly poorer Quality of Care (β = −0.50, *p* < 0.01) and Overall Satisfaction (β = −1.20, *p* < 0.01). Occupational status as a worker was strongly and positively associated with outcomes, especially Perception of Interaction (β = 3.53, *p* < 0.001) and Overall Satisfaction (β = 4.47, *p* < 0.001). Model fit indices indicated modest explanatory power, with R^2^ ranging from 0.09 (Similarity) to 0.20 (Overall Satisfaction).

The multivariable models, including interaction terms, revealed several significant predictors of patients’ perceptions of telemedicine. Adequate technical support was consistently associated with lower scores across all outcomes (e.g., Quality of Care, β = −2.50, *p* < 0.001; Total, β = −9.73, *p* < 0.001). In contrast, greater ease of technology use was positively associated with perceptions (e.g., Quality of Care, β = 1.40, *p* < 0.001; Perception of Interaction, β = 1.93, *p* < 0.001). Significant interaction effects indicated that the benefits of technology usability were reduced for patients at their first telemedicine visit (Quality of Care: β = −0.86, *p* < 0.001; Perception of Interaction: β = −0.51, *p* = 0.017; Total: β = −1.84, *p* < 0.001). Similarly, older age attenuated the positive association between ease of technology use and outcomes (Similarity: β = −0.02, *p* = 0.021; Total: β = −0.04, *p* = 0.048). In addition, male gender was positively associated with higher ratings (e.g., Quality of Care, β = 0.34, *p* = 0.006; Total, β = 1.24, *p* < 0.001), while higher educational level was modestly linked with better Quality of Care (β = 0.28, *p* = 0.038).

## 4. Discussion

The present study aimed to evaluate satisfaction and its predictors among patients with IBD who used telemedicine services. Overall, satisfaction levels were high across all TSQ domains, indicating broad acceptance of the remote care model within this context. However, the analysis revealed that satisfaction is multifactorial, largely mediated by the technological setting, individual patient characteristics, and clinical complexity, rather than by demographic or simple clinical variables alone.

Ease of use of the technology emerged as the most robust and consistent positive predictor. This finding is entirely consistent with meta-analyses that identify usability as one of the factors most frequently associated with telemedicine satisfaction, underscoring the need to remove barriers to its full implementation [34]. Conversely, the observation that patients who required technical support reported significantly lower satisfaction scores prompts reflection on an often-overlooked aspect: the relationship between the need for assistance and digital vulnerability [35]. In other words, satisfaction depends not only on service efficiency but also on patient predisposition [14] and pre-existing technological competence [36].

The finding that patients attending their first visit preferred face-to-face encounters over telemedicine adds another layer of complexity. Many patients favor in-person visits as their first contact with healthcare professionals [37]; this may be explained by a cultural factor, whereby patients perceive greater attention and engagement when interactions occur in person [38], preserving the perceived sense of being cared for that is often associated with traditional encounters [39], compared to telemedicine.

Patients’ clinical history and disease type also influenced satisfaction levels with telemedicine in our sample. Specifically, patients with CD reported lower satisfaction than those with UC [40,41]. This may stem from the fact that CD often requires objective evaluations that cannot be performed entirely remotely, as noted by Stidham et al. (2016) [42]. However, this hypothesis has not yet been demonstrated. In contrast, evidence from other chronic conditions suggests that telemedicine is more readily accepted in stable or less complex disease states [43].

Advanced age showed a dual effect: on the one hand, it correlated positively with overall satisfaction, likely due to logistical advantages and reduced travel; this is consistent with recent evidence from studies conducted during the COVID-19 pandemic, which demonstrated that telemedicine, despite age-related barriers, is an effective strategy enabling elderly patients to feel continuously supported in their therapeutic journey [25,44,45,46]. On the other hand, advanced age attenuated the positive impact of technological ease of use, suggesting that older age and its perceived limitations may reduce engagement with telemedicine [43], even when access is optimized.

Finally, the association between higher satisfaction and employment status observed in our sample may reflect a potential socioeconomic bias. Although telemedicine is a valuable strategy for keeping patients engaged in their care pathways and may improve health outcomes, it may also favor individuals with greater digital resources, thereby potentially exacerbating health inequalities across different populations [47]. This phenomenon is consistent with the concept of the digital divide, whereby differences in access to and familiarity with digital technologies can translate into unequal opportunities to benefit from telemedicine services [44,45,46,47,48].

Male patients reported slightly higher satisfaction with telemedicine than female patients [49]. This difference may reflect variations in expectations toward healthcare interactions, perceived relational needs, or confidence in using digital technologies [50]. Women may place greater emphasis on interpersonal communication, which can be perceived as less prominent in remote encounters [51]. Although the effect size was modest, future studies should further investigate sex- and gender-related determinants of telemedicine experience.

Our findings align with evidence from other contexts of chronic disease management. For instance, in type 2 diabetes, systematic reviews have demonstrated that telemedicine can enhance glycemic control and treatment adherence [42], while also reducing the utilization of traditional healthcare services [13], ultimately leading to improved outcomes. However, long-term satisfaction with telemedicine depends not only on such improvements but also on usability and the continuity of feedback from healthcare professionals [22]. Encouraging results regarding patient satisfaction with telemedicine have also been observed among patients with chronic cardiac conditions [52]. Indeed, telemedicine, especially when implemented through a multidisciplinary approach, may facilitate the management of complex patients, allow close monitoring of disease progression, and enhance patient engagement in their care pathway, thereby increasing satisfaction [53,54].

Several studies have also explored the use of telemedicine in oncology, reporting high levels of patient satisfaction and trust, which emphasizes the need to design systems that prioritize ease of use [55,56]. International comparative analyses have shown that patients in countries with more advanced digital infrastructure report significantly higher satisfaction levels than those in middle- or low-income countries, confirming the role of socio-technological factors [57]. Overall, the evidence suggests that satisfaction is a trans-clinical variable that can influence health outcomes but is itself influenced by non-clinical factors such as usability, technical support, perceived safety, and continuity of the patient–provider relationship.

Although satisfaction is an essential indicator of service acceptability, it does not necessarily equate to clinical effectiveness. The modest explanatory power of our models (R^2^ ≈ 0.20) suggests that unmeasured factors, such as empathy, trust in the healthcare provider, communication quality, and perceived continuity of care, may play a substantial role. Indeed, qualitative studies in other chronic conditions have confirmed that emotional reassurance and a sense of control influence satisfaction beyond technical aspects [13,58].

### 4.1. Strengths and Limitations

The strengths of the present study include a large sample size, clinical heterogeneity among participants, and the use of a validated instrument, I-TSQ. However, the cross-sectional design does not allow for causal inferences or assessments of long-term sustainability. Accordingly, the associations observed between technological or organisational factors (e.g., ease of use, platform access) and telemedicine satisfaction should not be interpreted as evidence of a direct causal relationship. Patients who are more satisfied with telemedicine may also be more inclined to perceive technology as easy to use, suggesting potential reverse or bidirectional associations.

Furthermore, satisfaction was self-reported, which may introduce social desirability bias. The absence of objective clinical indicators (e.g., remission, hospitalizations, adherence) limits the ability to correlate perceived satisfaction with measurable outcomes. Additionally, differences in organizational models and telemedicine workflows across centres, as well as limited socio-economic diversity, may limit the applicability of these findings to broader patient populations and less-structured healthcare settings. Finally, recruitment from specialized centers and the high educational level of participants may have overestimated the actual acceptability of telemedicine in the general population.

Although the multicentre design included geographically distinct areas of Italy, the study was not powered to perform region-specific analyses. Future studies involving a larger number of centres per region could explore potential geographical differences in telemedicine satisfaction related to infrastructure, digital literacy, and organizational models.

### 4.2. Practical Implications and Future Perspectives

The success of telemedicine depends as much on its technological design as on its human and organizational components. Integrating principles of user-centered design, pre-visit training, and hybrid care pathways can enhance patient satisfaction and engagement.

Longitudinal and randomized studies are needed to analyze the relationship between satisfaction, adherence, and clinical outcomes. Mixed-method investigations could further clarify how psychological factors, such as self-care, trust, and perceived control, mediate the relationship between telemedicine use and its perceived benefits.

Future research should also explore how advances in new technologies, including more intuitive platforms, artificial intelligence–driven support, and integrated digital tools, can be leveraged to improve telemedicine accessibility, patient engagement, and care personalization in IDB and chronic care in general [58,59].

Finally, health policies should incorporate digital equity measures to ensure that telemedicine services are accessible to all populations, preventing any form of inequality.

## 5. Conclusions

Telemedicine in IBD care is widely accepted and appreciated, even within this patient population; however, it remains vulnerable to inequalities and usability limitations. The interaction among digital literacy, prior experience, and clinical complexity largely shapes perceptions and the service’s usability.

To translate patient satisfaction into tangible clinical value, it is necessary to develop personalized models that account for age, disease type, and digital skills, while simultaneously ensuring relational continuity, proactive technical support, and digital inclusion.

Adapting telemedicine strategies to incorporate new technologies will be essential for maintaining patient satisfaction, enhancing usability, and ensuring equitable care across diverse organizational and socio-economic contexts.

## Figures and Tables

**Table 1:** Descriptives results.

Variable	Value
**Gender n (%)**	
Female	369 (52.3)
Male	336 (47.7)
**Marital status n (%)**	
Divorced	135 (19.1)
Single	150 (21.3)
Married	336 (47.7)
Widowed	84 (11.9)
**Educational level n (%)**	
University degree	164 (23.3)
Lower secondary (middle school licence)	68 (9.6)
Master’s/PhD	74 (10.5)
No schooling	1 (0.1)
Primary school	6 (0.9)
Middle school	80 (11.3)
Upper secondary (high school)	312 (44.3)
**Occupation n (%)**	
Homemaker	20 (2.8)
Unemployed	134 (19.0)
Employed	340 (48.2)
Retired	101 (14.3)
Student	110 (15.6)
**Pathology n (%)**	
Ulcerative Colitis	387 (54.9)
Crohn’s Disease	318 (45.1)
**Disease duration n (%)**	
≤1 year	21 (3.0)
1–5 years	92 (13.0)
6–10 years	202 (28.7)
>10 years	390 (55.3)
**Treatment n (%)**	
Biologics	433 (61.4)
Surgery	24 (3.4)
Non-biologic therapy	107 (15.2)
Immunosuppressants	98 (13.9)
Immunosuppressants + Biologics	19 (2.7)
Immunosuppressants + Surgery	4 (0.6)
5-ASA (Mesalazine) only	10 (1.4)
5-ASA + Surgery	8 (1.1)
5-ASA and steroids	2 (0.3)
**Symptoms n (%)**	
Fatigue	195 (27.6)
Diarrhoea	97 (13.8)
Abdominal pain and diarrhoea	131 (18.6)
None	193 (27.4)
Weight loss	23 (3.3)
Rectal bleeding	66 (9.4)
**Caregiver**	
No	154 (21.9)
Yes	551 (78.2)
**Reason for televisit**	
Other	92 (13.0)
New symptoms/clinical deterioration	169 (24.0)
Therapeutic plan renewal	189 (26.8)
Review of blood test results	255 (36.2)
**First televisit**	
No	283 (40.1)
Yes	422 (59.9)
**Ease of accessing the televisit**	
Difficult	5 (0.7)
Easy	314 (44.5)
Very difficult	9 (1.3)
Very easy	327 (46.4)
Neither easy nor difficult	50 (7.1)
**Ease of using the required technology**	
Difficult	7 (1.0)
Easy	315 (44.7)
Very easy	340 (48.2)
Neither easy nor difficult	43 (6.1)
**Received adequate technical support**	
No	7 (1.0)
Yes	698 (99.0)
**Televisit contribution to disease management**	
Little	8 (1.1)
Moderate	368 (52.2)
High	151 (21.4)
Neither high nor low	177 (25.1)
**Televisit allowed more frequent contact with healthcare provider**	
No	146 (20.7)
Yes	559 (79.3)
**Time saved compared with in-person visits**	
Little	3 (0.4)
Moderate	277 (39.3)
High	418 (59.3)
Neither high nor low	7 (1.0)

Legend. %: percentage, n: number, 5-ASA: 5-aminosalicylic acid.

**Table 2:** Descriptive statistics of Telemedicine Satisfaction outcomes.

Outcome	Mean (SD)	Range
Quality of Care	16.85 (1.78)	11–20
Similarity	20.21 (2.48)	11–25
Perception of Interaction	16.59 (1.94)	8–20
Overall Satisfaction	57.50 (4.93)	35–70

Legend: SD = standard deviation.

**Table 3:** Predictors of TSQ domains and overall score.

Predictors	Quality of Care Beta (SE)	Similarity Beta (SE)	Perception of Interaction Beta (SE)	Overall Satisfaction Beta (SE)
Intercept	17.87 (0.87) ***	24.36 (1.25) ***	16.61 (0.92) ***	61.95 (2.32) ***
Technical Support (Yes)	−2.75 (0.65) ***	−4.60 (0.93) ***	−2.54 (0.69) ***	−10.04 (1.73) ***
Occupation (Worker)	1.43 (0.44) ***	0.003 (0.18)	3.53 (0.55) ***	4.47 (0.33) ***
Age (years)	0.005 (0.004)	0.007 (0.006)	0.016 (0.004) ***	0.026 (0.011) *
First Time Televisit	−0.66 (0.14) ***	−0.60 (0.20) **	−0.69 (0.15) ***	−1.98 (0.37) ***
Pathology (Crohn’s disease)	−0.41 (0.13) **	−0.27 (0.18)	−0.64 (0.14) ***	−1.48 (0.34) ***
More Frequent Televisit	0.37 (0.18) *	0.17 (0.25)	0.89 (0.18) ***	1.98 (0.47) ***
Gender (Male)	0.35 (0.13) **	0.56 (0.18) **	0.32 (0.13) *	1.20 (0.34) ***
Access (Likert 1–5)	−0.24 (0.10) *	−0.35 (0.14) *	−0.41 (0.10) ***	−0.84 (0.26) **
TechUse (Likert 1–5)	0.59 (0.11) ***	0.41 (0.16) *	0.77 (0.12) ***	1.75 (0.30) ***
Ed.Level (High education)	0.28 (0.13) *	−0.17 (0.19)	−0.06 (0.14)	0.14 (0.36)
Treatment (Immunosuppressants)	−0.09 (0.18)	−0.04 (0.26)	−0.18 (0.19)	−0.48 (0.49)
Treatment (Non-Biologic Therapy)	−0.35 (0.18)	−0.50 (0.26)	−0.37 (0.19)	−1.33 (0.48) **
Therapy (Subcutaneous)	−0.50 (0.17) **	0.01 (0.34)	−0.08 (0.09)	−1.20 (0.42) **
F (df) & R^2^	F = 9.45 (12, 692), R^2^ = 0.14	F = 5.51 (12, 692), R^2^ = 0.09	13.3 (12, 692), R^2^ = 0.19	14.75 (12, 692), R^2^ = 0.20

Notes: Bold terms are significant; levels are indicated as follows: *** *p* < 0.001, ** *p* < 0.01, * *p* < 0.05. Legends: SE = standard error; df = degrees of freedom; R^2^ = variance explained.

## Data Availability

The datasets used and/or analysed during the current study are available from the corresponding author on reasonable request.

## Supplementary Materials

The following supporting information can be downloaded at: https://www.mdpi.com/article/10.3390/medicina62010147/s1, File S1, Strobe checklist.

## Abbreviations

The following abbreviations are used in this manuscript:

Acronym	Meaning
5-ASA	5-aminosalicylic acid
CD	Crohn’s disease
CI	Confidence interval
CFI	Comparative Fit Index
COVID-19	Coronavirus disease 2019
HbA1c	Haemoglobin A1c
IBD	Inflammatory bowel disease
I-TSQ	Italian Telemedicine Satisfaction Questionnaire
IQR	Interquartile range
MDES	Minimum detectable effect size
RMSEA	Root Mean Square Error of Approximation
SD	Standard deviation
SE	Standard error
TSQ	Telemedicine Satisfaction Questionnaire
UC	Ulcerative colitis
U-healthcare	Ubiquitous healthcare
VIF	Variance inflation factor
